# Transcription Profile of Aging and Cognition-Related Genes in the Medial Prefrontal Cortex

**DOI:** 10.3389/fnagi.2016.00113

**Published:** 2016-05-17

**Authors:** Lara Ianov, Asha Rani, Blanca S. Beas, Ashok Kumar, Thomas C. Foster

**Affiliations:** ^1^Department of Neuroscience, McKnight Brain Institute, University of FloridaGainesville, FL, USA; ^2^Genetics and Genomics Program, Genetics Institute, University of FloridaGainesville, FL, USA

**Keywords:** aging, cognitive flexibility, prefrontal cortex, set shifting task, transcription

## Abstract

Cognitive function depends on transcription; however, there is little information linking altered gene expression to impaired prefrontal cortex function during aging. Young and aged F344 rats were characterized on attentional set shift and spatial memory tasks. Transcriptional differences associated with age and cognition were examined using RNA sequencing to construct transcriptomic profiles for the medial prefrontal cortex (mPFC), white matter, and region CA1 of the hippocampus. The results indicate regional differences in vulnerability to aging. Age-related gene expression in the mPFC was similar to, though less robust than, changes in the dorsolateral PFC of aging humans suggesting that aging processes may be similar. Importantly, the pattern of transcription associated with aging did not predict cognitive decline. Rather, increased mPFC expression of genes involved in regulation of transcription, including transcription factors that regulate the strength of excitatory and inhibitory inputs, and neural activity-related immediate-early genes was observed in aged animals that exhibit delayed set shift behavior. The specificity of impairment on a mPFC-dependent task, associated with a particular mPFC transcriptional profile indicates that impaired executive function involves altered transcriptional regulation and neural activity/plasticity processes that are distinct from that described for impaired hippocampal function.

## Introduction

The extent to which cognition declines over the course of aging varies across individuals. Neuroimaging studies indicate that the pattern of cognitive decline is related to changes in the structure and activity of the prefrontal cortex (PFC) and hippocampus (Grady et al., 2005; Persson et al., 2006; Dennis et al., 2008; Park and Reuter-Lorenz, 2009; Migo et al., 2016), suggesting that individual differences in cognitive aging may result from vulnerability and reorganization of these neural systems. In addition, altered white matter integrity could influence connectivity of the PFC with other brain regions (O'Sullivan et al., 2001; Pfefferbaum et al., 2005; Salat et al., 2005; Andrews-Hanna et al., 2007; Bennett et al., 2011; Borghesani et al., 2013). The molecular mechanisms for vulnerability and adaptive reorganization during aging have been the subject of speculation (Jackson et al., 2009; Kumar et al., 2009; McEwen and Morrison, 2013; Gray and Barnes, 2015). Previous work using microarray technology indicates that over the course of aging, the transcription of genes linked to inflammation and synaptic function increases and decreases, respectively, within a number of brain regions (Prolla, 2002; Blalock et al., 2003; Verbitsky et al., 2004; Erraji-Benchekroun et al., 2005; Loerch et al., 2008; Burger, 2010; Bordner et al., 2011; Haberman et al., 2011; VanGuilder et al., 2011; Zeier et al., 2011; Cribbs et al., 2012; Yuan et al., 2012; Berchtold et al., 2013; Primiani et al., 2014), suggesting possible mechanisms for variability in cognitive decline.

While it is widely thought that transcription is linked to cognitive function, there is relatively little information on the PFC transcriptional profile, which attempts to link altered gene expression to an age-related decline in behaviors that depend on the PFC. Indeed, the PFC provides several unique challenges for examining the relationship of transcription to age-related cognitive impairment. The PFC can be divided into several sub-regions and there is a long-standing debate over the equivalence of anatomical regions within the PFC across species (Uylings and van Eden, 1990; Preuss, 1995; Brown and Bowman, 2002; Vertes, 2004; Hoover and Vertes, 2007). In addition, the PFC is involved in executive function, which encompasses a number of cognitive processes including attention, response inhibition, working memory, and mental flexibility (Robbins, 1996; Bizon et al., 2012).

In the current study, we exploit individual differences in behavior to examine the relationship between age-related changes in cognition and transcription. Young and aged rats were characterized on two tasks that are age-sensitive, including an attentional set shift task that depends on the mPFC (Brown and Bowman, 2002; Kesner and Churchwell, 2011) and on a hippocampal-dependent spatial episodic memory task (Foster, 2012; Foster et al., 2012). RNA sequencing (RNA-seq) was used to construct transcriptomic profiles for the mPFC, white matter, and CA1 region of the hippocampus. Expression differences associated with aging and cognition, defined by variability in set shift or spatial memory behavior, were examined. Finally, the aging and cognition mPFC gene sets were compared to microarray data from other studies to test specific hypotheses. The results indicate that expression of immediate-early genes (IEGs) related to neural activity and synaptic plasticity decline with age in the mPFC; however, within the group of aged animals, expression of IEGs is up regulated in animals that exhibit delayed set shift behavior.

## Methods

### Animals

Procedures involving animal subjects have been reviewed and approved by the Institutional Animal Care and Use Committee and were in accordance with guidelines established by the U.S. Public Health Service Policy on Humane Care and Use of Laboratory. Male Fischer 344 rats of two ages, young (5–6 months, *n* = 11) and aged (17–22 months, *n* = 20) were obtained from National Institute on Aging colony (Taconic) through the University of Florida Animal Care and Service facility. Animals were maintained on a 12:12 h light schedule, and provided ad lib access to food and water prior to the set shifting task.

### Behavioral studies

#### Set shifting operant task

##### Apparatus

Testing in the set shifting task was conducted in standard rat behavioral test chambers (30.5 × 25.4 × 30.5 cm, Coulbourn Instruments, Whitehall, PA) with metal front and back walls, transparent Plexiglas side walls, and a floor composed of steel rods (0.4 cm in diameter) spaced 1.1 cm apart. Each test chamber was housed in a sound-attenuating cubicle, and was equipped with a recessed food pellet delivery trough located 2 cm above the floor in the center of the front wall. The trough was fitted with a photobeam to detect head entries and a 1.12 W lamp for illumination. Food rewards consisted of one 45 mg grain-based food pellet for each correct response (PJAI, Test Diet, Richmond, IN). Two retractable levers were located to the left and right of the food trough (11 cm above the floor), and a 1.12 W cue lamp was located 3.8 cm above each lever. An additional 1.12 W house light was mounted near the top of the rear wall of the sound-attenuating cubicle. An activity monitor was positioned above each test chamber to monitor locomotor activity throughout each session. This monitor consisted of an array of infrared (body heat) detectors focused over the entire test chamber. Movement in the test chamber (in x, y, or z planes) was defined as a relative change in the infrared energy falling on the different detectors. A computer interfaced with the behavioral test chambers and equipped with Graphic State 3.01 software (Coulbourn Instruments) was used to control experiments and collect data.

##### Behavioral shaping

The design of the set shifting task was based previously published methods (Floresco et al., 2008; Beas et al., 2013). Prior to the start of behavioral testing, rats were reduced to 85% of their free feeding weights over the course of 5 days and maintained at this weight for the duration of the experiments. Rats were trained and tested in the same behavioral testing chamber during the course of the experiment. Rats progressed through four stages of shaping prior to the start of the set shifting task, with new stages beginning on the day immediately following completion of the previous stage. On the day prior to Shaping Stage 1, each rat was given five 45 mg food pellets in its home cage to reduce neophobia to the food reward used in the task. Shaping Stage 1 consisted of a 64-min session of magazine training, involving 38 deliveries of a single food pellet with an inter-trial interval (ITI) of 100 ± 40 s. Shaping Stage 2 consisted of lever press training, in which a single lever (left or right, counterbalanced across groups) was extended and a press resulted in delivery of a single food pellet. After reaching a criterion of 50 lever presses in 30 min, rats were then trained on the opposite lever using the same procedures.

Shaping Stage 3 consisted of 90 trials that were designed to train rats to press the levers after their insertion into the test chamber. Each 20 s trial began with illumination of the house light and insertion of a single lever (either left or right, randomly selected within each pair of trials) into the test chamber where it remained for a maximum of 10 s. A response on the lever within this time window resulted in retraction of the lever, delivery of a single food pellet, and continued illumination of the house light for an additional 4 s. If a rat failed to respond on the lever within 10 s, the lever was retracted and the house light turned off, and the trial was scored as an omission. Rats received at least 4 daily sessions in this stage, and were trained until reaching criterion performance of fewer than 10 omissions out of the 90 trials.

Shaping Stage 4 was designed to determine each rat's side bias (i.e., preference for one lever over the other). Each trial consisted of multiple phases. In the first phase of a trial, the house light was illuminated and both levers were inserted into the test chamber. A response on either lever resulted in retraction of both levers and delivery of a single food pellet. In the second phase of a trial, both levers were again inserted, but only a response on the lever opposite to that chosen in the first phase resulted in food delivery. A response on the same lever chosen in the first phase (i.e., “incorrect”) resulted in the levers being retracted and the house light being extinguished. After a “correct” response in this second phase of a trial, a new trial was initiated, whereas after an “incorrect” response, the second phase of the trial was repeated. The second phase was repeated until rats made a “correct” response. The session ended after a total of 45 completed trials. The side associated with the greatest number of total responses across this phase of testing was considered a rat's biased side.

##### Visual cue discrimination

Following shaping stage 4, rats were trained to press the lever signaled by the illumination of a cue light over the lever. Each 20 s trial began with illumination of one of the cue lights (left or right, randomly selected in each pair of trials). After 3 s, the house light was illuminated and both levers were inserted into the chamber (the cue light remained illuminated while the levers were extended). A response on the lever corresponding to the cue light (a correct response) resulted in the house light remaining on for 4 s, during which time the levers were retracted, the cue light was extinguished, and a single food pellet was delivered. A response on the opposite lever (an incorrect response) or failure to respond within 10 s (omission) resulted in retraction of both levers and all lights being extinguished. Rats were considered to have acquired the task upon reaching criterion performance of eight consecutive correct trials (and at least 30 total trials, excluding omissions), with the maximum number of trials per session set at 120. Rats that failed to acquire the task within a single session (young: *n* = 5; aged: *n* = 13) received additional sessions on subsequent days.

##### Left/right discrimination (set shift)

After reaching criterion performance on the visual cue discrimination, rats were tested the next day in the set shift condition, in which the task contingencies were altered. In this condition, rats were required to ignore the visual cue and instead to consistently choose the left or right lever (whichever was not their biased side as determined in Shaping Stage 4). Hence, accurate performance required rats to “shift” their attention away from the visual cue and toward the left/right position of the lever. Beyond the shift in reward contingencies, trials were identical in presentation to those in the visual cue discrimination (i.e., on each trial, both levers were presented; with the cue light illuminated over one lever). As in the visual cue discrimination, the location of the illuminated cue light was randomized (left or right) in each pair of trials. Rats were considered to have acquired the task upon reaching criterion performance of eight consecutive correct trials, excluding omissions. The maximum number of trials per session was set at 120 and all rats acquired the task within a single session.

#### Morris water maze

Following completion of set shifting, animals were again provided ad lib access to food and water for ~7 weeks prior to testing on the water maze. Animals were trained in a black tank, 1.7 m in diameter, positioned in a well-lit room. The pool was surrounded by black walls and black curtain. For spatial training an assortment of two- and three-dimensional cues were hung on the walls and curtain. Water (27 ± 2°C) was maintained at a level ~8 cm below the surface of the tank. Methods employed to assess sensory-motor deficits and impaired episodic spatial memory on the water maze have been published previously (Foster et al., 1991; Kumar and Foster, 2013; Guidi et al., 2014). For cue and spatial tasks, training consisted of five blocks with three trials per block and training on each task was massed into a single day. Inter-trial intervals were 20 s and inter-block intervals were ~15 min. Rats remained on the platform between trials and in home cages under the heat lamp after each block. Behavioral data was acquired with Noldus EthoVision computer tracking software (Noldus Information Technology, Leesburg, VA, USA) and included path-length and latency to escape to the platform, platform crossing and time in the goal and opposite quadrants.

Rats were first trained on the cue discrimination version of the water escape task. The escape platform was extended ~1 cm above the water level and a white Styrofoam flag was attached. For each trial, the platform position and start location were randomized. If an animal did not escape the water maze within 60 s, the rat was gently guided to the platform. Three days following cue training, animals were trained on the spatial discrimination task. For spatial discrimination, the escape platform was hidden ~1.5 cm beneath the water level and remained in the same location relative to the distal cues in the room for the duration of the initial spatial training. Fifteen minutes following the end of training on block 5, a free-swim probe trial was administered as a measure of learning. For the probe trial, the platform was removed and the animal placed in the tank for 60 s. A spatial discrimination index was computed according to the formula (G − O)/(G + O) where G and O represent the percent of time spent in the goal quadrant and quadrant opposite the goal, respectively.

##### Statistical analysis of behavior

The total numbers of trials required to achieve criterion (TTC) on the visual cue discrimination and on the left/right discrimination (set shift) were used as the indices of performance. Mean distance to find the platform during each training block for the water maze cue and spatial tasks and probe trial data, platform crossing, and discrimination index, were employed to examine learning on the water maze. For the distance measures, repeated measures analyses of variance (ANOVAs) were used to examine age and training effects. One way ANOVAs were used to examine aged effects for the water maze probe trial data and TTC measures from the operant tasks. Fisher's protected least significant difference comparisons, with the *p*-value set at 0.05, were used to localize differences.

#### Tissue collection

**Figure 1:**
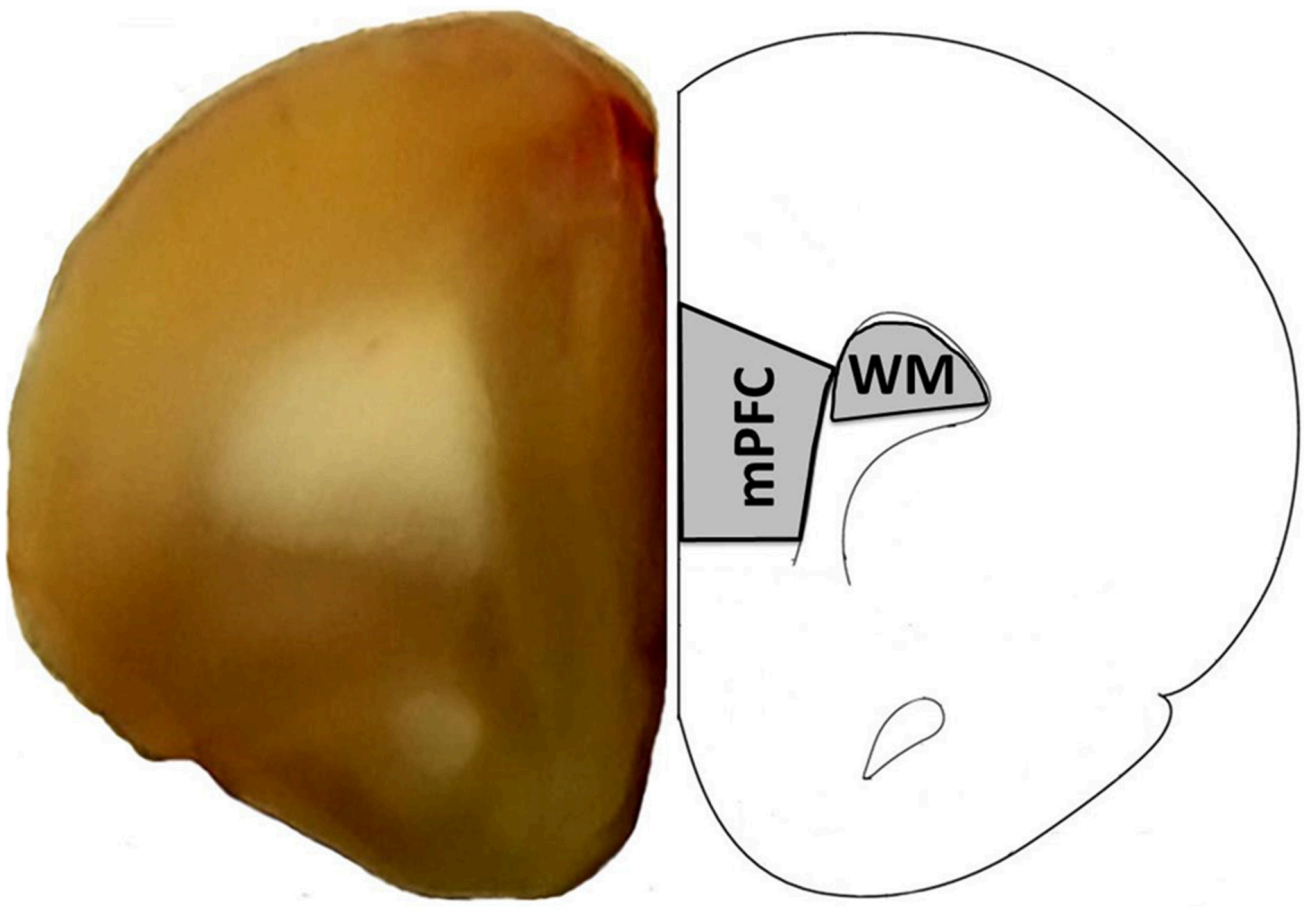
**Region of the mPFC and white matter (WM) collected for RNA-seq**. The right panel provides a schematic of a coronal slice +2.7 anterior to bregma diagram as adapted from Paxinos and Watson (1986) and illustrates the region of the mPFC and white matter collected for RNA-seq. The left panel shows a coronal slice from this same region.

Two weeks following water maze testing, rats were anesthetized with isoflurane (Piramal Healthcare), decapitated and the brain was rapidly removed. The PFC was blocked into 1 mm coronal slices. The mPFC including the prelimbic and infralimbic regions were collected from two sections (between +5.0 and +2.5 anterior to bregma; Paxinos and Watson, 1986). For a subset of animals (young = 8, aged = 9), white matter was collected adjacent to the mPFC (Figure 1). For region CA1, the hippocampus was isolated, a 1–2 mm slice was removed from the dorsal hippocampus, and the CA1 region was dissected (Blalock et al., 2003; Zeier et al., 2011). The collected tissue was immediately frozen in liquid nitrogen and stored in −80°C until processed.

#### RNA and library preparation

RNA was isolated from the mPFC, CA1, and white matter using the RNeasy Lipid Tissue Mini kit (Qiagen, catalog number 74804) and DNase digestion was performed with the RNase-Free DNase Set (Qiagen, catalog number 79254). The concentration was measured with the NanoDrop 2000 spectrophotometer and the RNA integrity number (RIN) was quantified by the University of Florida Interdisciplinary Center for Biotechnology Research using the High Sensitivity RNA Screen Tape in an Agilent 2200 Tapestation system. The average RIN across all regions was 8.02 (±SEM 0.05). External RNA Controls Consortium (ERCC) spike-in controls (Thermo Fisher, catalog number 4456740) were added to a subset of samples and the mRNA was selected with the Dynabeads mRNA DIRECT Micro kit (Thermo Fisher, catalog number 61021). Whole transcriptome libraries were prepared with the Ion Total RNA-seq Kit v2 (Thermo Fisher, catalog number 4475936) with the addition of the Ion Xpress barcodes for multiplex sequencing (Thermo Fisher, catalog number 4475485). The concentration of the libraries was quantified by the Qubit dsDNA HS Assay (Thermo Fisher, catalog number Q32851) and size distribution was evaluated with the High Sensitivity D1000 Screen Tape in the Tapestation system.

#### Reverse transcription quantitative polymerase chain reaction

Reverse transcription quantitative polymerase chain reaction (RT-qPCR) was performed in a subset of samples to validate RNA-seq results. cDNA was prepared using the QuantiTect Reverse Transcription kit (Qiagen, catalog number 205311) and quantitative PCR was completed with the TaqMan Gene Expression Assays (*Arc:* Rn00571208_g1*, Egr1:* Rn00561138_m1*, Egr2:* Rn00586224_m1*, Egr4:* Rn00569509_g1*, Fos:* Rn02396759_m1*, Lin7b:* Rn00572781_m1*, Gapdh:* Rn01775763_g1) in a 7300 Real-Time PCR system with SDS software version 1.3.1 (Applied Biosystems). The ΔΔCT method (Livak and Schmittgen, 2001) was used to determine the relative cDNA levels. Differences in the subset of RNA-seq and RT-qPCR were confirmed using *t*-tests between young and aged rats and between age impaired and unimpaired animals.

#### Sequencing, bioinformatics, and statistical analysis

Template preparation was performed in the Ion Chef system and sequencing was completed in the Ion Proton (Thermo Fisher). ERCC analysis was executed in the Torrent Server with the ERCC analysis plugin. Spiked samples contained R^2^ above 0.9 with at least 60 transcripts. On average, each sample contained 18.8 million reads of 131 base pair length. Low quality reads were removed from the FASTQ files and the data was aligned to the rn5 genome using the two step alignment method for Ion Proton transcriptome data with TopHat2 and Bowtie2 in the Partek Flow servers (Partek Inc.). Gene-level counts were generated from BAM files using the *featureCounts* function in the R package *Rsubread* (Liao et al., 2014). The Rnor_5.0.78.gtf file was used for annotation and count normalization was performed with the DESeq package in R. The data for this study has been uploaded to NCBI's Gene Expression Omnibus under the accession number: GSE75772.

Gene filtering and initial statistical analysis was performed according to our previously published work (Blalock et al., 2003; Aenlle et al., 2009; Aenlle and Foster, 2010; Zeier et al., 2011). Gene lists were initially filtered to remove those genes with counts of 5 or less, which resulted in the detection of over 18,000 Ensembl database genes from each area. Gene lists were further filtered such that only genes with annotation of at least one gene ontology (GO) term were considered for gene enrichment analysis. Filtering for GO terms resulted in 15075 mPFC genes, 15235 genes, and 15084 white matter genes. For differential expression analysis associated with age, a statistical filter was performed in each tissue type independently, using a one-way ANOVA generated in Partek Genomics Suite 6.6, with *p* < 0.025 according to our previous work (Blalock et al., 2003; Aenlle et al., 2009; Aenlle and Foster, 2010; Zeier et al., 2011). For examination of mPFC gene expression related to cognition, Pearson's correlations were calculated between the behavioral TTC measures for the set shifting task or discrimination index score for spatial learning and the expression of each gene in the mPFC transcriptome. Correlations were limited to aged animals in order to remove age as a confound. Due to multiple comparisons, the confidence in any single gene is low; therefore, gene enrichment analysis was performed under the assumption that changes in biological process with age or cognition would result in a shift in the expression of clusters of genes related to the biological process. For gene enrichment and functional annotation clustering analysis, data sets of genes that exhibited an increase or decrease in expression were separately submitted to the NIH database for annotation, visualization, and integrated discovery (DAVID; Huang et al., 2007a,b). Enrichment analysis was limited to gene ontology for biological processes and cellular components with the Benjamini False Discovery Rate (FDR) *p* < 0.05 as a cut-off for cluster selection. The heat map were generated in Partek Genomics Suite 6.6 using the genes that were identified in DAVID with counts which were standardized to z-scores. In other cases, specific hypotheses were tested by comparing gene expression with previous published work using microarrays. In this case, we determined whether the previously published genes were detected by our procedures. This set of genes that were common across studies represented the total data set. Next, we used a fold change and chi squared test to determine if the genes were altered in the same direction, relative to what would be expected by chance. The number of genes that were significantly altered in the same direction was determined using one-tailed *t*-tests with the direction specified by the microarray studies. A FDR was calculated by calculating the number of genes expected to change in a specified direction using the formula T^*^p, where T = total number of genes tested and p is the significance level (0.05). The expected number of genes was divided by the number of genes that were significantly different in the predicted direction to obtain the FDR.

## Results

### Behavior

#### Set shift

**Figure 2:**
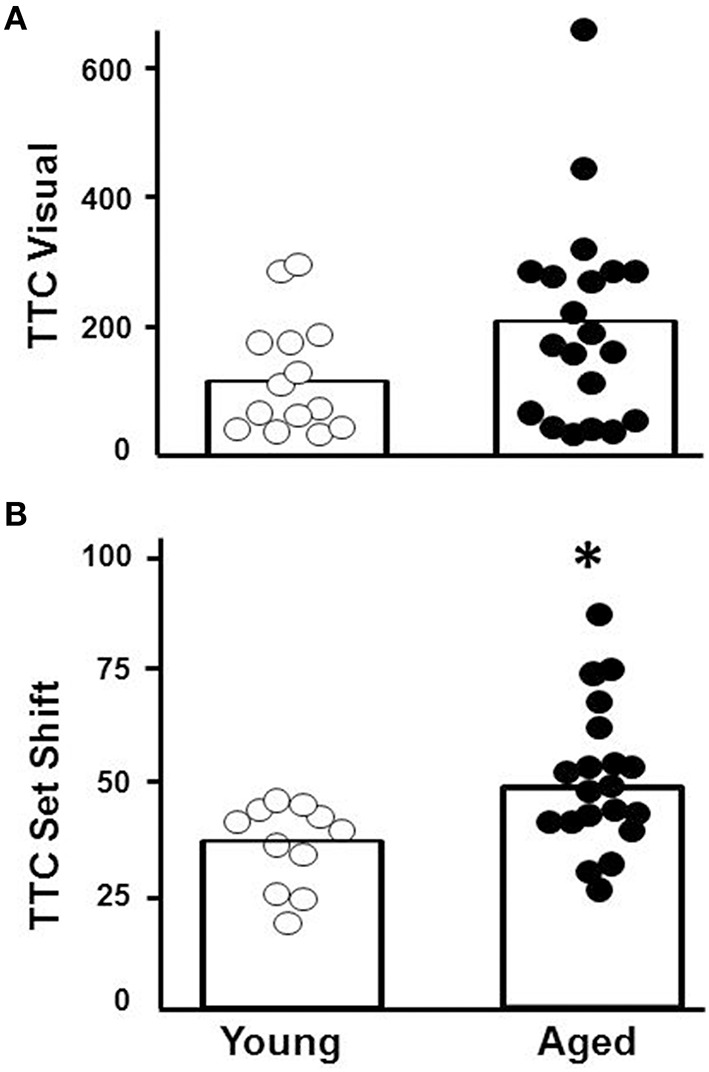
**Performance on the visual discrimination and set shift operant tasks**. Trials to criteria (TTC) are illustrated for individual aged (filled circles, *n* = 20) and young (open circles, *n* = 11) animals during performance of the **(A)** initial visual discrimination and **(B)** set shift tasks. Asterisk indicates that aged animals exhibited more trails to criteria for the set shift task (*p* < 0.01). The open bars indicate the mean TTC for each group.

Young (*n* = 11) and aged (*n* = 20) animals were trained on the visual discrimination operant task followed by set shift testing. Aged animals exhibited considerable variability in performance on each task (Figure 2), with some aged animals performing in a range similar to young. Examination of the TTC for visual discrimination indicated no effect of age [*F*_(1, 29)_ = 2.15, *p* = 0.15; Figure 2A]. In contrast, an age difference in TTC was observed for the set shifting (left/right discrimination) behavior [*F*_(1, 29)_ = 7.95, *p* < 0.01], with a subset of aged rats exhibiting an increase in TTC relative to young (Figure 2B).

#### Water maze

**Figure 3:**
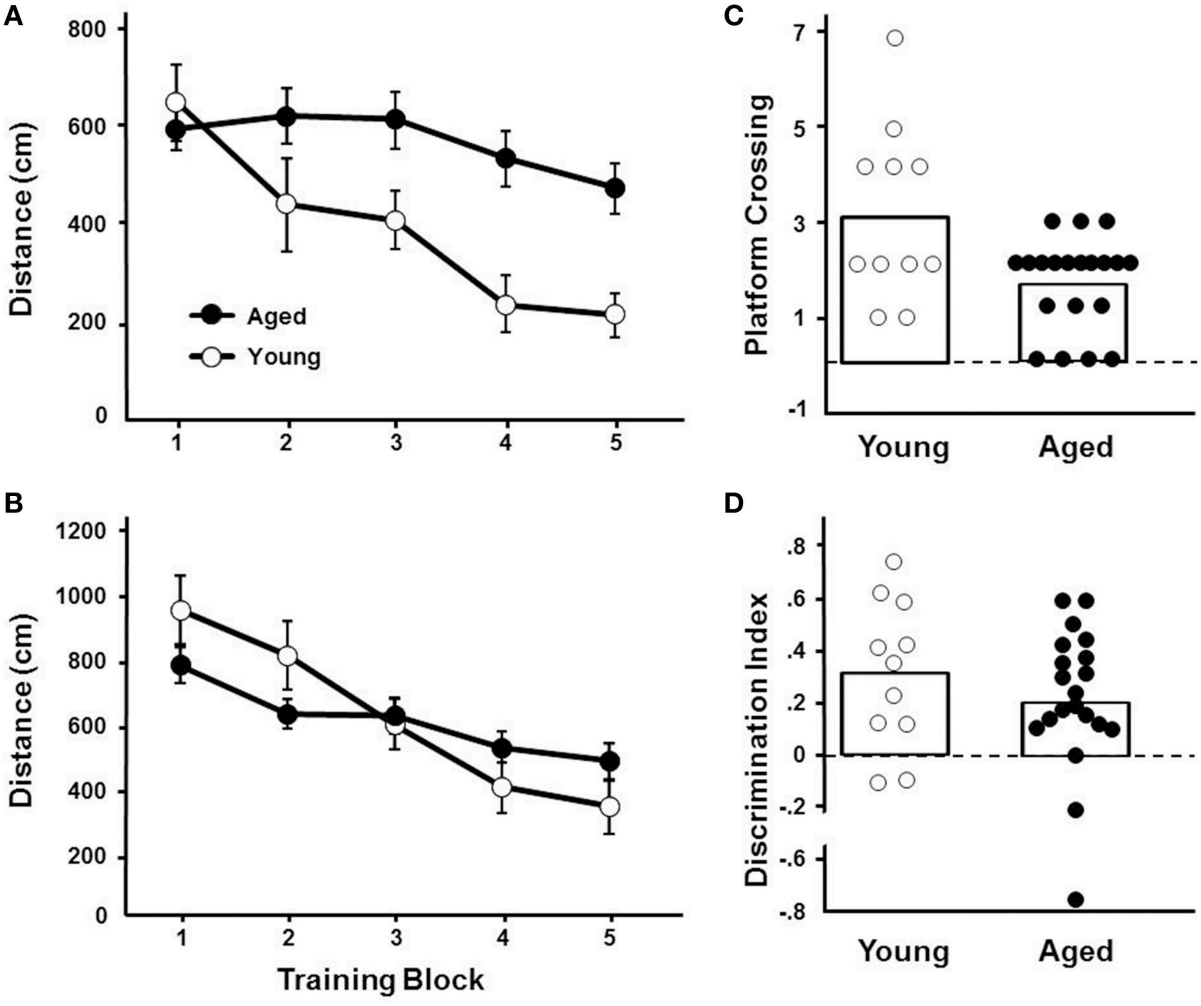
**Performance on the water maze task**. Symbols indicate the mean (±SEM) escape path length to the escape platform during five training blocks on the **(A)** cue and **(B)** spatial discrimination tasks for young (open symbols) and aged (filled symbols) animals. Individual **(C)** platform crossing and **(D)** discrimination index scores for young (open symbols) and aged (filled symbols) animals. The open bars indicate the means for each group.

For the cue discrimination version of the water maze task, all animals were able to find the visible platform during the 60 s time limit during the last three trials (block 5). A repeated measures ANOVA for the cue discrimination task indicated an effect of training [*F*_(4, 116)_ = 7.84, *p* < 0.0001] and age [*F*_(1, 29)_ = 11.43, *p* < 0.005] and an interaction of age and training [*F*_(4, 116)_ = 2.92, *p* < 0.05] due to superior performance by young animals during the final training blocks (Figure 3A). A repeated measures ANOVA for the spatial discrimination task indicated an effect of training [*F*_(4, 116)_ = 17.82, *p* < 0.0001] and an interaction of age and training [*F*_(4, 116)_ = 3.13, *p* < 0.05; Figure 3B]. The results of the probe trial indicated a decrease in platform crossings for aged animals [*F*_(1, 29)_ = 8.52, *p* < 0.01; Figure 3C]. No age effect was observed for the discrimination index; however, consistent with previous reports (Blalock et al., 2003; Foster, 2012; Kumar and Foster, 2013; Guidi et al., 2014), there was considerable variability (Figure 3D). For aged animals, the relationship between behavioral measures on the operant tasks (visual discrimination TTC, set shift TTC) and on the water maze (distance to escape on block 5 of the cue task, distance to escape on block 5 of the spatial task, platform crossings, and discrimination index of the probe trial) was examined (Table 1). The results indicated a correlation between the distance on block 5 of the spatial task and the discrimination index (*r* = −0.484, *p* < 0.05), such that longer escape distances on the spatial task were associated with poorer discrimination index scores.

**Table 1:** **Behavioral correlations**.

	**Visual TTC**	**Cue block 5**	**Spatial block 5**	**Crossings**	**Discrimination index**
Set shift TTC	0.168	0.115	−0.356	0.145	0.154
Visual TTC		0.245	−0.185	0.197	0.309
Cue block 5			0.29	−0.126	0.17
Spatial block 5				−0.376	−0.484^*^
Crossings					0.311

* *p < 0.05*.

### Gene expression

#### Gene expression related to aging

**Figure 4:**
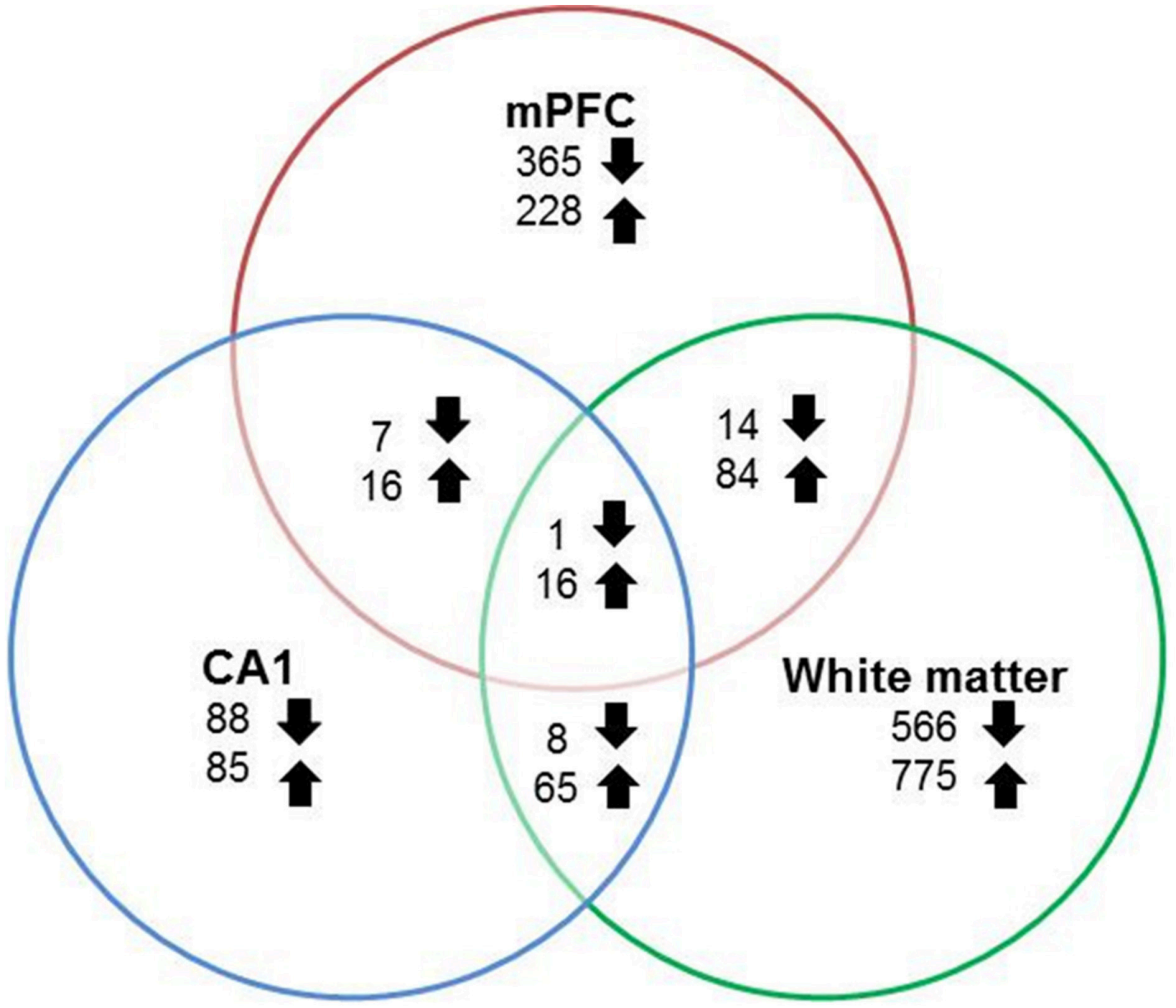
**Number of genes altered during aging across regions**. Graphic summary of the total number of genes whose expression either significantly (*p* < 0.025) increased (up arrow) or decreased (down arrow) in the mPFC, region CA1, white matter, and across regions.

RNA-seq libraries from the mPFC and region CA1 of the hippocampus were prepared from all animals (young = 11, aged = 20). In addition, libraries were prepared from white matter, collected from a subset of animals (young = 8, aged = 9). Gene expression data was statistically filtered for age differences using ANOVAs for each tissue type with a cut-off set at *p* < 0.025. The largest and smallest number of age-related genes was observed for white matter (1529 genes) and region CA1 (286 genes), with 731 genes altered in the mPFC. Although, expression changes were relatively distinct, there was some overlap. Furthermore, for genes that overlapped across any two regions, the number of up regulated genes was 2–8 times larger than genes that decreased expression (Figure 4).

Based on functional studies, it has been suggested that the mPFC of the rat may be analogous to the dorsolateral PFC of humans and non-human primates (Dias et al., 1997; Birrell and Brown, 2000; Kesner and Churchwell, 2011). To determine possible expression changes in the rat mPFC that may correspond to changes observed in humans, we compared our results with a microarray study examining age-related changes in the dorsolateral (Brodmann's area 9) PFC of humans (Erraji-Benchekroun et al., 2005). For the 414 age-related probes from the dorsolateral PFC of the original human data set, we were able to identify 318 rat genes in the mPFC and the direction of change, increasing or decreasing, was determined for the rat. From the set of 318 genes, we observed 203 genes (64%) that changed in the same direction as predicted in humans and a chi square analysis indicated that the directional changes were different than that expected by chance (*x*^2^ = 11.86, *p* < 0.001). One-tailed *t*-tests (*p* < 0.05 with direction specified by age-related changes in humans) were conducted to examine age differences in expression of these 318 genes. A total of 60 genes (39 decreased, 21 increased) reached significance (FDR: 0.27; Table 2).

**Table 2:** **Age-related changes across the rat mPFC and the dorsolateral PFC of humans**.

**Gene symbol**	**Gene name**	**Direction**
*Anp32b*	Acidic leucine-rich nuclear phosphoprotein 32 family member B	Increased
*C4a*	Complement component 4A	Increased
*Chi3l1*	Chitinase 3-like 1 (cartilage glycoprotein-39)	Increased
*Clu*	Clusterin	Increased
*Daam2*	Disheveled associated activator of morphogenesis 2	Increased
*Enah*	Enabled homolog	Increased
*Fgfr1*	Fibroblast growth factor receptor 1	Increased
*Gfap*	Glial fibrillary acidic protein	Increased
*Hipk2*	Homeodomain interacting protein kinase 2	Increased
*Maob*	Monoamine oxidase B	Increased
*Map4*	Microtubule-associated protein 4	Increased
*Map7*	Microtubule-associated protein 7	Increased
*Mid1ip1*	MID1 interacting protein 1	Increased
*Moxd1*	Monooxygenase, DBH-like 1	Increased
*Mxi1*	MAX interactor 1	Increased
*Plekhb1*	Pleckstrin homology domain containing, family B (evectins) member 1	Increased
*Ptk2b*	PTK2B protein tyrosine kinase 2 beta	Increased
*Rassf2*	Ras association (RalGDS/AF-6) domain family member 2	Increased
*Ssfa2*	Sperm specific antigen 2	Increased
*Sun2*	Unc-84 homolog B (*C. elegans*)	Increased
*Zcchc24*	Zinc finger, CCHC domain containing 24	Increased
*Adamts8*	ADAM metallopeptidase with thrombospondin type 1 motif, 8	Decreased
*Agfg1*	ArfGAP with FG repeats 1	Decreased
*Cacna1g*	Calcium channel, voltage-dependent, T type, alpha 1G subunit	Decreased
*Cask*	Calcium/calmodulin-dependent serine protein kinase (MAGUK family)	Decreased
*Cdh11*	Cadherin 11, type 2, OB-cadherin (osteoblast)	Decreased
*Cdh8*	Cadherin 8, type 2	Decreased
*Cdk5*	Cyclin-dependent kinase 5	Decreased
*Crh*	Corticotropin releasing hormone	Decreased
*Crhr1*	Corticotropin releasing hormone receptor 1	Decreased
*Cx3cl1*	Chemokine (C-X3-C motif) ligand 1	Decreased
*Cyp26b1*	Cytochrome P450, family 26, subfamily B, polypeptide 1	Decreased
*Dcaf7*	WD repeat domain 68	Decreased
*Dnajb5*	DnaJ (Hsp40) homolog, subfamily B, member 5	Decreased
*Dusp14*	Dual specificity phosphatase 14	Decreased
*Edn3*	Endothelin 3	Decreased
*Egr4*	Early growth response 4	Decreased
*Eif4g1*	Eukaryotic translation initiation factor 4 gamma, 1	Decreased
*Fam131a*	Family with sequence similarity 131, member A	Decreased
*Fam49a*	Family with sequence similarity 49, member A	Decreased
*Gabra4*	Gamma-aminobutyric acid (GABA) A receptor, alpha 4	Decreased
*Gng4*	Guanine nucleotide binding protein (G protein), gamma 4	Decreased
*Grm2*	Glutamate receptor, metabotropic 2	Decreased
*Hmgcs1*	3-hydroxy-3-methylglutaryl-Coenzyme A synthase 1	Decreased
*Htr2a*	5-hydroxytryptamine (serotonin) receptor 2A	Decreased
*Kcnf1*	Potassium voltage-gated channel, subfamily F, member 1	Decreased
*Kcnh1*	Potassium voltage-gated channel, subfamily H (eag-related), member 1	Decreased
*Lancl2*	LanC lantibiotic synthetase component C-like 2 (bacterial)	Decreased
*Large*	Like-glycosyltransferase	Decreased
*Lppr4*	Plasticity related gene 1	Decreased
*Mapk4*	Mitogen-activated protein kinase 4	Decreased
*Mmd*	Monocyte to macrophage differentiation-associated	Decreased
*Neto2*	Neuropilin (NRP) and tolloid (TLL)-like 2	Decreased
*Rprm*	Reprimo, TP53 dependent G2 arrest mediator candidate	Decreased
*Sel1l3*	KIAA0746 protein	Decreased
*Slc8a2*	Solute carrier family 8 (sodium/calcium exchanger), member 2	Decreased
*Sst*	Somatostatin	Decreased
*Sstr1*	Somatostatin receptor 1	Decreased
*St8sia3*	ST8 alpha-N-acetyl-neuraminide alpha-2,8-sialyltransferase 3	Decreased
*Trib2*	Tribbles homolog 2	Decreased

The data sets for differentially expressed up regulated and down regulated genes were separately submitted to NIH DAVID for enrichment analysis based on gene ontology for biological processes and cellular components. Cluster selection cut-off was restricted to clusters with a Benjamini FDR *p* < 0.05. In each case, several genes were observed in multiple related clusters, which are shown below.

##### Increased expression

**Figure 5:**
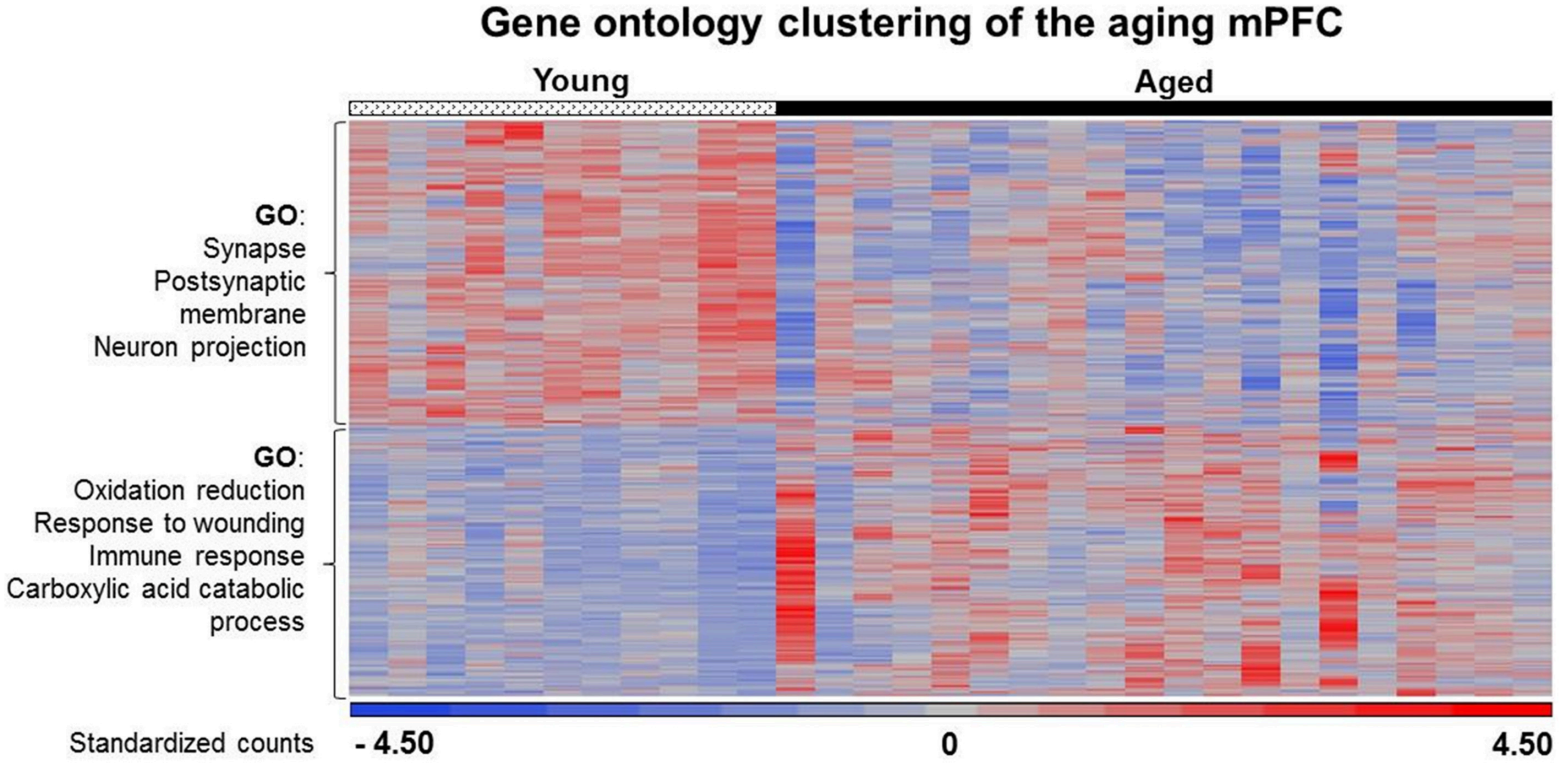
**Heat map of age-related changes in gene expression for the mPFC**. Each row represents a differentially expressed gene (*p* < 0.025) associated with aging. Expression for each gene was converted to a standardized score and the color represents the standard deviation increasing (red) or decreasing (blue) relative to the mean (gray). The age-related gene enrichment clusters are indicated (FDR *p* < 0.05). Top clusters: Genes that exhibit a decrease from young (left) to aged (right) animals. Bottom: Genes that exhibit an increase from young to aged animals.

In general, age-related changes in transcription involve up regulation of genes linked to immune response, oxidative stress, and the lysosome (Lee et al., 2000; Blalock et al., 2003; Verbitsky et al., 2004; Fraser et al., 2005; Rowe et al., 2007; de Magalhães et al., 2009; Kadish et al., 2009; Lipinski et al., 2010; VanGuilder et al., 2011; Zeier et al., 2011; Yuan et al., 2012). Expression of immune response related genes was particularly evident in the white matter and mPFC. For the 940 white matter genes, enrichment was observed for immune response (GO:0006955, 85 genes, FDR *p* = 4.1^−25^), defense response (GO:0006952, 70 genes, FDR *p* = 1.3^−16^), and the lysosome (GO:0005764, 33 genes, FDR *p* = 2.2^−9^). Similarly, for the 344 genes that increased expression in the mPFC, gene enrichment was observed for biological processes linked to oxidation reduction (GO:0055114, 30 genes, FDR *p* = 0.02), response to wounding (GO:0009611, 24 genes, FDR *p* = 0.02), and adaptive immune response (GO:0002250, 10 genes, FDR *p* = 0.01; Table 3). Increased expression in the mPFC was also observed for carboxylic and catabolic process (GO:0046395, 13 genes, FDR *p* = 0.002; Figure 5). Finally, for 182 CA1 genes, enrichment was observed for the lysosome (GO:0005764, 10 genes, FDR *p* = 0.007). Up regulation was observed for response to wounding (GO:0009611, 11 genes, FDR *p* = 0.38) and defense response (GO:0006952, 10 genes, FDR *p* = 0.44); however, the clusters did not reach the FDR cut-off.

**Table 3:** **Increased mPFC expression during aging**.

**Gene symbol**	**Gene name**	**Response to wounding**	**Immune response**	**Oxidation reduction**
*Adam17*	ADAM metallopeptidase domain 17	X	X	
*Fcgr2b*	Fc fragment of IgG, low affinity IIb, receptor (CD32)	X	X	
*Pou2f3*	POU class 2 homeobox 3	X		
*Rab27a*	RAB27A, member RAS oncogene family	X	X	
*Timp3*	TIMP metallopeptidase inhibitor 3	X		
*Bmp6*	Bone morphogenetic protein 6	X		
*Ctsb*	Cathepsin B	X		
*Clu*	Clusterin	X		
*F11*	Coagulation factor XI	X		
*C1qc*	Complement component 1, q subcomponent, C chain	X	X	
*C1qb*	Complement component 1, q subcomponent	X	X	
*C4b*	Complement component 4B	X	X	
*Fn1*	Fibronectin 1	X		
*Gfap*	Glial fibrillary acidic protein	X		
*Hmox1*	Heme oxygenase (decycling) 1	X		X
*Itgb2*	Integrin beta 2	X		
*Ncf1*	Neutrophil cytosolic factor 1	X		
*Pecam1*	Platelet/endothelial cell adhesion molecule 1	X		
*Pdpn*	Podoplanin	X		
*Sparc*	Secreted protein, acidic, cysteine-rich	X		
*Serpina1*	Serpin peptidase inhibitor, clade A	X		
*Treml1*	Triggering receptor expressed on myeloid cells-like 1	X		
*Tp73*	Tumor protein p73	X		
*Erbb2*	v-erb-b2 erythroblastic leukemia viral oncogene homolog 2	X		
*Swap70*	SWAP-70 protein		X	
*Il18*	Interleukin 18		X	
*Il18bp*	Interleukin 18 binding protein		X	
*Lilrb3*	Leukocyte immunoglobulin-like receptor		X	
*Hpd*	4-hydroxyphenylpyruvate dioxygenase			X
*Acadm*	Acyl-Coenzyme A dehydrogenase			X
*Acadl*	Acyl-Coenzyme A dehydrogenase, long-chain			X
*Adhfe1*	Alcohol dehydrogenase, iron containing, 1			X
*Aldh1l1*	Aldehyde dehydrogenase 1 family			X
*Aldh2*	Aldehyde dehydrogenase 2 family			X
*Aldh6a1*	Aldehyde dehydrogenase 6 family, member A1			X
*Akr1c13*	Aldo-keto reductase family 1, member C13			X
*Aass*	Aminoadipate-semialdehyde synthase			X
*Aifm3*	Apoptosis-inducing factor			X
*Cdo1*	Cysteine dioxygenase, type I			X
*Cybrd1*	Cytochrome b reductase 1			X
*Dcxr*	Dicarbonyl L-xylulose reductase			X
*Fmo2*	Flavin containing monooxygenase 2			X
*Hadh*	Hydroxyacyl-Coenzyme A dehydrogenase			X
*Hadha*	Hydroxyacyl-Coenzyme A dehydrogenase			X
*Hsd17b4*	Hydroxysteroid (17-beta) dehydrogenase 4			X
*Idh2*	Isocitrate dehydrogenase 2 (NADP+)			X
*Maob*	Monoamine oxidase B			X
*Acad11*	Acyl-Coenzyme A dehydrogenase family			X
*Phyhd1*	Phytanoyl-CoA dioxygenase domain containing 1			X
*Plod1*	Procollagen-lysine 1, 2-oxoglutarate 5-dioxygenase 1			X
*Pyroxd2*	Pyridine nucleotide-disulphide oxidoreductase domain 2			X
*Phgdh*	Phosphoglycerate dehydrogenase			X
*Prodh*	Proline dehydrogenase			X
*Slc14a1*	Solute carrier family 14 (urea transporter)			X
*Tbxas1*	Thromboxane A synthase 1, platelet			X
*Tph1*	Tryptophan hydroxylase 1			X
*Xdh*	Xanthine dehydrogenase			X

##### Decreased expression

Previous work indicates that aging is associated with decreased expression of genes linked to neuronal/synaptic genes (Blalock et al., 2003; Lu et al., 2004; Verbitsky et al., 2004; Burger et al., 2007; Aenlle and Foster, 2010; Zeier et al., 2011; Berchtold et al., 2013; Primiani et al., 2014). In the case of the 589 genes that were decreased in white matter, enrichment was observed for cell division (GO:0051301, 16 genes, FDR *p* = 0.04). For the 104 genes that decreased in area CA1, clustering did not pass our cut-off. For the 387 mPFC genes that decreased with age, common genes were observed for clusters related to the synapse (GO:0045202, 25 genes, FDR *p* = 1.2^−4^) and postsynaptic membrane (GO:0045211, 14 genes, FDR *p* = 4.1^−4^; Table 4). In addition, decreased expression was observed for neuron projection (GO:0043005, 22 genes, FDR *p* = 0.015; Figure 5). Thus, the age-related decrease in neuronal genes was particularly evident in the mPFC.

**Table 4:** **Decreased mPFC expression during aging**.

**Gene symbol**	**Gene name**	**Synapse**	**Postsynaptic membrane**	**Neuron projection**
*Anks1b*	Ankyrin repeat and sterile alpha motif domain containing 1B	X	X	X
*Clstn3*	Calsyntenin 3	X	X	
*Cbln1*	Cerebellin 1 precursor	X		
*Chrm2*	Cholinergic receptor, muscarinic 2	X	X	X
*Chrna5*	Cholinergic receptor, nicotinic, alpha 5	X	X	X
*Cyp19a1*	Cytochrome P450, family 19, subfamily a, polypeptide 1	X		X
*Doc2a*	Double C2-like domains, alpha	X		
*Dnm3*	Dynamin 3	X		X
*Gabra4*	Gamma-aminobutyric acid (GABA) A receptor, alpha 4	X	X	
*Gad1*	Glutamate decarboxylase 1	X		X
*Grip1*	Glutamate receptor interacting protein 1	X	X	
*Grid1*	Glutamate receptor, ionotropic, delta 1	X	X	
*Grid2*	Glutamate receptor, ionotropic, delta 2	X	X	
*Grik3*	Glutamate receptor, ionotropic, kainate 3	X	X	X
*Grm7*	Glutamate receptor, metabotropic 7	X	X	X
*Grm8*	Glutamate receptor, metabotropic 8	X	X	X
*Glrb*	Glycine receptor, beta	X	X	
*Lin7b*	Lin-7 homolog b (*C. elegans*)	X	X	
*Magee1*	Melanoma antigen, family E, 1	X	X	X
*Scamp1*	Secretory carrier membrane protein 1	X		
*Prkaca*	Similar to CG2662-PA; protein kinase, cAMP-dependent, catalytic, alpha	X		
*Slc2a3*	Solute carrier family 2 (facilitated glucose transporter), member 3	X		
*Sv2b*	Synaptic vesicle glycoprotein 2b	X		
*Syt6*	Synaptotagmin VI	X		
*Ywhaz*	Tyrosine 3-monooxygenase/tryptophan 5-monooxygenase activation protein, zeta polypeptide	X		
*Bace1*	Beta-site APP cleaving enzyme 1			X
*Dcc*	Deleted in colorectal carcinoma			X
*Dpysl2*	Dihydropyrimidinase-like 2			X
*Dpysl5*	Dihydropyrimidinase-like 5			X
*Dctn2*	Dynactin 2			X
*Got1*	Glutamic-oxaloacetic transaminase 1			X
*Klhl1*	Kelch-like 1 (Drosophila)			X
*Map2k4*	Mitogen activated protein kinase kinase 4			X
*Kcnj12*	Potassium inwardly-rectifying channel			X
*Pgr*	Progesterone receptor			X
*Ptprn2*	Protein tyrosine phosphatase			X
*Tacr3*	Tachykinin receptor 3			X

#### mPFC gene expression related to behavior

**Figure 6:**
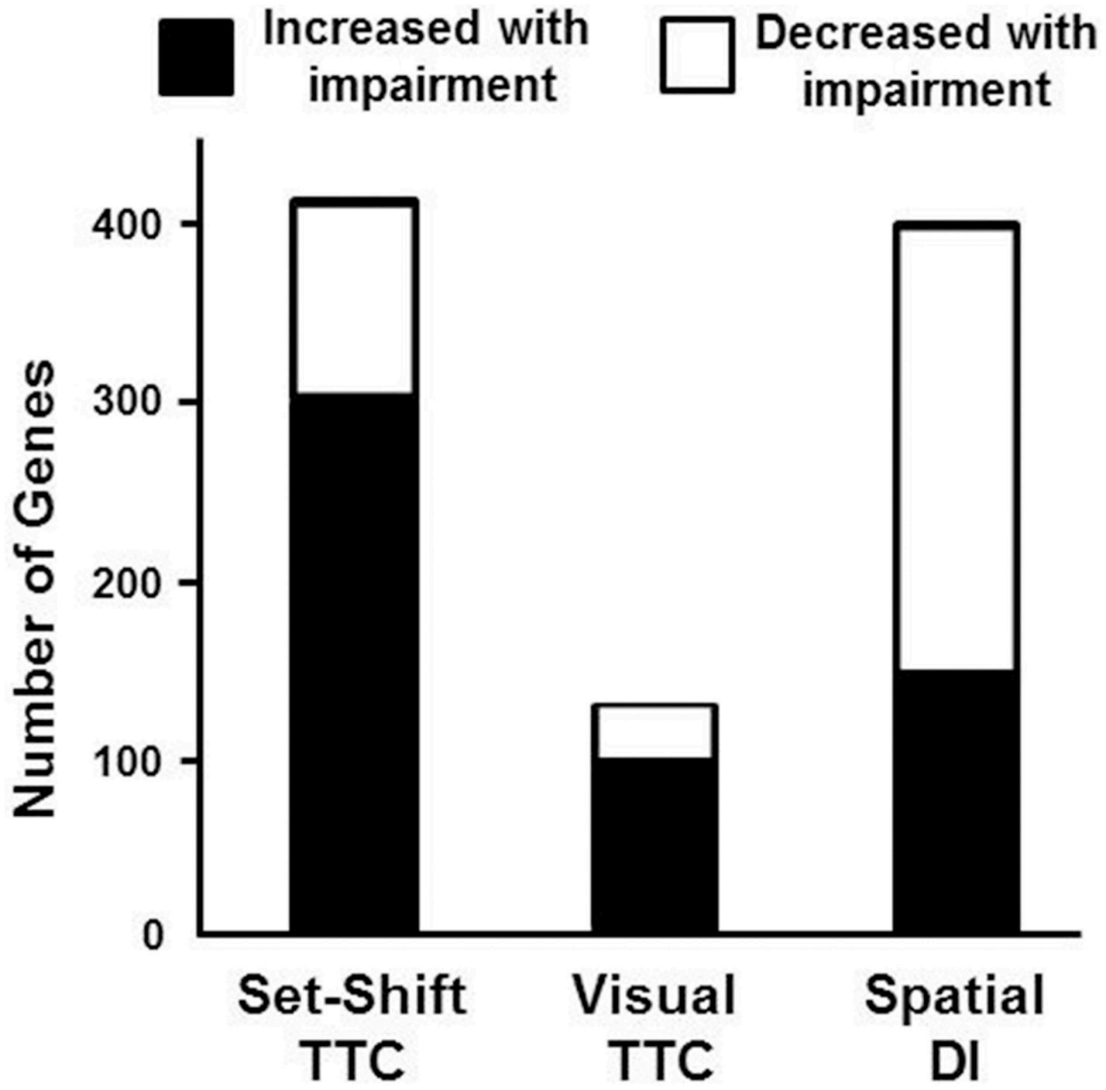
**Number of genes correlated with behavioral measures**. Graphic summary for the number of mPFC genes whose expression either significantly increased (filled) or decreased (open) in relation to performance on the set shift and visual discrimination operant tasks, and a spatial memory task (Pearson's correlation *p* < 0.025). A large number of genes exhibited increased expression associated with impaired set shifting compared to visual discrimination and spatial discrimination index (DI).

To examine behavioral specificity of transcriptional changes, Pearson's correlations were run comparing expression of mPFC genes with the TTC for set shifting and visual discrimination, and the discrimination index score for the water maze. Correlations were limited to aged animals in order to remove age as a confound and correlations were performed across all genes in the mPFC transcriptome. Using a cut-off set at *p* < 0.025 (*r* = 0.499), a total of 416 genes were correlated with behavioral flexibility. Most of the genes (73%) were positively correlated with the set shift TTC score (303 genes increasing expression with impairment), with 113 genes negatively correlated (decreased in impaired animals). A similar analysis for the visual discrimination TTC indicated many fewer mPFC genes (135 total) correlated with acquisition of the visual discrimination, with 103 mPFC genes positively correlated (increased in animals with more trials to criteria) and 32 genes that were negatively correlated (decreased in animals with more trials to criteria). Unlike the set shift behavior, more mPFC genes exhibited decreased expression (62%) in animals with poorer spatial learning. A total of 401 mPFC genes correlated with the water maze discrimination index, with 249 genes positively correlated with the discrimination index (decreased in animals with poor spatial learning) and 152 mPFC genes negatively correlated (increased in spatial learning impaired animals; Figure 6).

**Figure 7:**
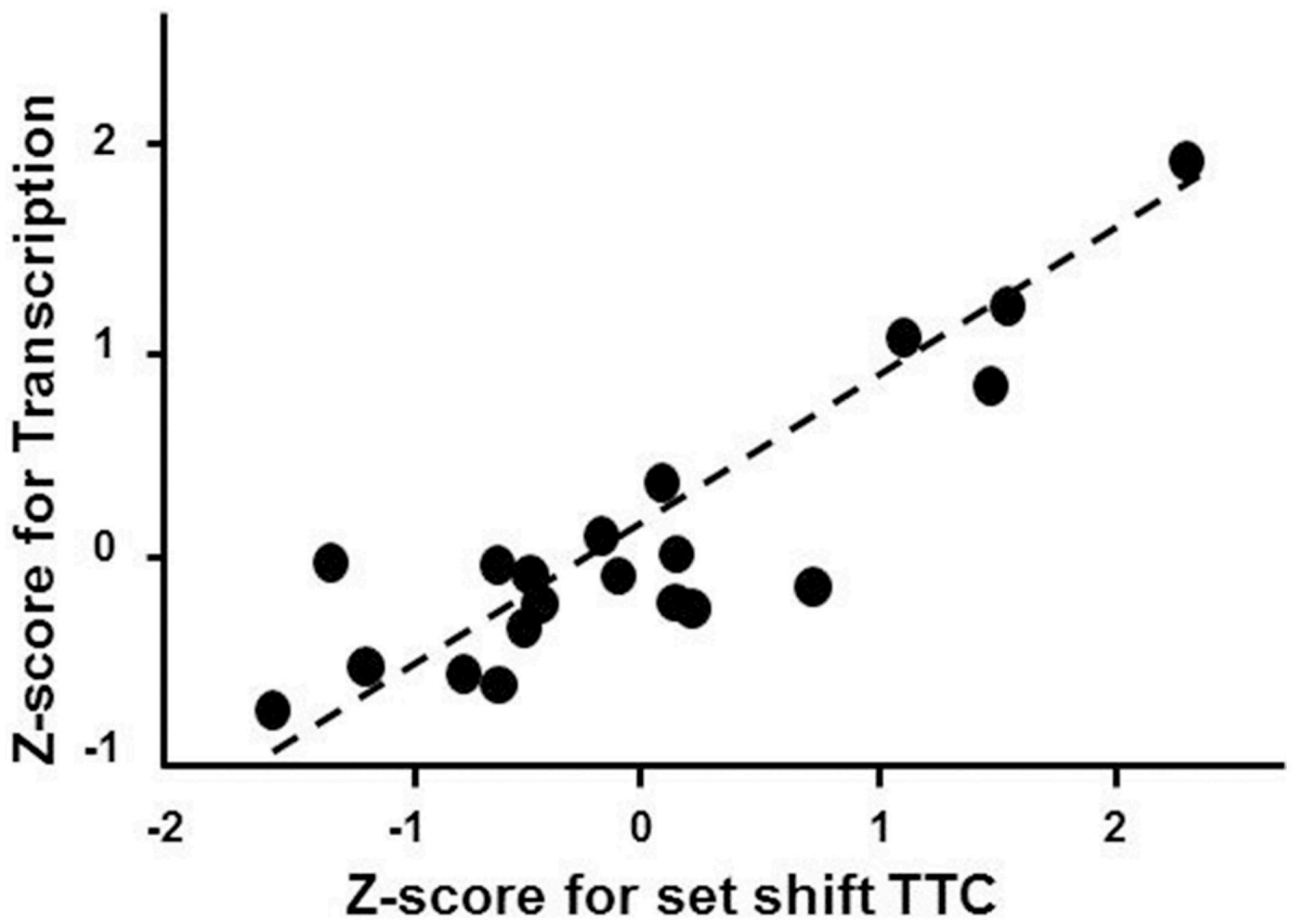
**Impaired set shifting is associated with increased expression of genes involved in transcription regulation**. The z-scores for the cluster of 46 transcription regulation genes were averaged for each animal. Individual mean z-scores (y-axes) are plotted relative to z-scores for set shift TTC (x-axes). The correlation is illustrated as a regression line (dashed line).

Gene enrichment analysis was conducted to determine which mPFC genes and biological processes might be good markers for the age-related impairment in set shift behavior. For mPFC genes that were negatively correlated with the set shift TTC (decreased expression in impaired animals that delay shifting), no gene enrichment clusters were observed to pass the cut-off for cluster selection. For gene expression that positively correlated with the TTC score (increasing in animals that delayed shifting), clusters were observed for response to organic substance (GO:0010033, 34 genes, FDR *p* = 0.01), regulation of apoptosis (GO:0042981, 27 genes, FDR *p* = 0.02) and the regulation of transcription (GO:0045449, 46 genes, FDR *p* = 0.027), which contained the largest number of genes (Table 5). An index score describing expression of transcription regulation genes was generated for each animal by first standardizing the expression of the 46 transcription genes and the standard scores were averaged within each animal. The standard scores were plotted against the standardized TTC set shift score (*r* = 0.89) to illustrate the correspondence of transcription regulators with set shift behavior (Figure 7). Interestingly, several IEGs linked to neuronal activity (*Arc, Egr1, Egr2, Egr3, Egr4, Fos, Fosb, Fosl2, Junb*) were observed to increase in association with delayed set shift behavior.

**Table 5:** **Positive correlation of mPFC genes with set shift TTC**.

**Gene symbol**	**Gene name**	**Regulation of transcription**	**Response to organic substance**	**Regulation of apoptosis**
*Bcl6b*	B-cell CLL/lymphoma 6, member B	X		
*Bcor*	BCL6 co-repressor	X		
*Cebpb*	CCAAT/enhancer binding protein (C/EBP), beta	X		
*Cebpd*	CCAAT/enhancer binding protein (C/EBP), delta	X		X
*Dnajb5*	DnaJ (Hsp40) homolog, subfamily B, member 5	X	X	
*Fos*	FBJ osteosarcoma oncogene	X	X	
*Fosb*	FBJ osteosarcoma oncogene B	X	X	
*Jun*	Jun oncogene	X	X	X
*Mxd3*	Max dimerization protein 3	X		
*Meis1*	Meis homeobox 1	X		
*Nab2*	Ngfi-A binding protein 2	X		
*Smarcal1*	Swi/SNF related matrix associated	X		
*Tsc22d3*	TSC22 domain family, member 3	X		X
*Ccdc101*	Coiled-coil domain containing 101	X		
*Cry2*	Cryptochrome 2 (photolyase-like)	X		
*Csrnp1*	Cysteine-serine-rich nuclear protein 1	X		
*Dlx1*	Distal-less homeobox 1	X		X
*Egr1*	Early growth response 1	X	X	
*Egr2*	Early growth response 2	X	X	
*Egr3*	Early growth response 3	X		
*Egr4*	Early growth response 4	X		
*Fosl2*	Fos-like antigen 2	X	X	
*Hes3*	Hairy and enhancer of split 3 (Drosophila)	X		
*Hmox1*	Heme oxygenase (decycling) 1	X	X	X
*Ing3*	Inhibitor of growth family, member 3	X		X
*Eomes*	Integrin alpha 9; eomesodermin homolog	X		
*Kdm6b*	Jumonji domain containing 3	X		
*Jmjd6*	Jumonji domain containing 6	X		
*Junb*	Jun B proto-oncogene	X	X	
*Mnt*	Max binding protein	X		X
*Mef2b*	Myocyte enhancer factor 2B	X		
*Npas1*	Neuronal PAS domain protein 1	X		
*Npas4*	Neuronal PAS domain protein 4	X		
*Nfkbia*	Nuclear factor of kappa light polypeptide gene enhancer	X	X	X
*Nr4a1*	Nuclear receptor subfamily 4, group A, member 1	X		X
*Nr4a2*	Nuclear receptor subfamily 4, group A, member 2	X	X	X
*Pax1*	Paired box 1	X		
*Per1*	Period homolog 1 (Drosophila)	X		
*Rxra*	Retinoid X receptor alpha	X	X	X
*Srf*	Serum response factor (c-fos transcription factor)	X	X	
*Sim2*	Single-minded homolog 2	X		
*Timeless*	Timeless homolog	X		
*Tle3*	Transducin-like enhancer of split 3	X		
*Trib1*	Tribbles homolog 1	X	X	
*Mafk*	v-maf musculoaponeurotic fibrosarcoma oncogene	X		
*Mycn*	v-myc myelocytomatosis viral related oncogene	X		
*Cuzd1*	CUB and zona pellucida-like domains 1		X	
*Rerg*	RAS-like, estrogen-regulated, growth-inhibitor		X	
*Apob*	Apolipoprotein B (including Ag(x) antigen)		X	
*Cpn1*	Carboxypeptidase N, polypeptide 1		X	
*Cdkn1a*	Cyclin-dependent kinase inhibitor 1A		X	X
*Dlc1*	Deleted in liver cancer 1		X	X
*Dusp4*	Dual specificity phosphatase 4		X	
*Gng7*	Guanine nucleotide binding protein (G protein), gamma 7		X	
*Hspa1b, Hspa1a*	Heat shock 70 kD protein 1B, 1A		X	X
*Hsd11b2*	Hydroxysteroid 11-beta dehydrogenase 2		X	
*Irs2*	Insulin receptor substrate 2		X	
*Lats2*	Large tumor suppressor 2		X	
*Plat*	Plasminogen activator, tissue		X	
*Kcna5*	Potassium voltage-gated channel, member 5		X	
*Ppp5c*	Protein phosphatase 5, catalytic subunit		X	
*Slc6a3*	Solute carrier family 6, member 3		X	
*Sphk1*	Sphingosine kinase 1		X	X
*Sts*	Steroid sulfatase		X	
*Socs3*	Suppressor of cytokine signaling 3		X	
*Vamp2*	Vesicle-associated membrane protein 2		X	
*Bard1*	BRCA1 associated RING domain 1			X
*Bag3*	Bcl2-associated athanogene 3			X
*Gfral*	GDNF family receptor alpha like			X
*Nuak2*	NUAK family, SNF1-like kinase, 2			X
*Aifm3*	Apoptosis-inducing factor, mitochondrion-associated 3			X
*Cidea*	Cell death-inducing DNA fragmentation factor			X
*C5*	Complement component 5			X
*Grm4*	Glutamate receptor, metabotropic 4			X
*Pim1*	Pim-1 oncogene			X
*Plekhf1*	Pleckstrin homology domain containing, family F			X
*Serinc3*	Serine incorporator 3			X
*Lck*	Lymphocyte-specific protein tyrosine kinase			X

Next we compared our results to a previously published microarray study that examined gene expression across rat species that differ on tests of attention (Qiu et al., 2010). The spontaneously hypertensive-rat (SHR), a well characterized model of impaired attention, exhibiting impairment on the five-choice serial reaction time task and attentional set shift relative to the Wistar-Kyoto rat (De Bruin et al., 2003; Sagvolden et al., 2005). Using RNA-seq, we were able to detect 48 genes that were correlated with set shift behavior and were previously reported to differ in the mPFC of SHR and Wistar-Kyoto rats (Qiu et al., 2010). In order to compare our data with the results of Qiu et al. (2010), a mean split for the set shift TTC scores of aged animals (mean TTC = 51.7) was used to separate aged animals into those that delayed shifting and were considered aged-impaired (AI) and aged-unimpaired (AU). One-tailed *t*-tests were run on the 48 genes using a *p* < 0.05 and the direction specified by the results of Qiu et al. (2010). For the 26 genes that were predicted to increase, 16 genes exhibited a significant increase (FDR: 0.08; Table 6). Interestingly, for the 16 genes that exhibited a significant increase in expression in AI rats, these genes also exhibited decreased expression during aging with seven genes (*Arc, Egr1, Egr3, Egr4, Junb, Klf10, Nr4a3*) exhibiting a significant (*p* < 0.05) decrease with age. Finally, for the 22 genes that were previously reported to decrease expression in SHR animals, no genes were significantly decreased in AI animals compared to AU animals.

**Table 6:** **Increased expression in the mPFC for AI vs. AU**.

**Gene symbol**	**Gene name**	**AI vs. AU fold**	**Age fold**
*Arc*	Activity regulated cytoskeletal-associated protein	1.89	−1.19
*Bhlhe40*	Basic helix-loop-helix domain containing, class B2	1.22	−1.08
*Btg2*	B-cell translocation gene 2, anti-proliferative	1.49	−1.28
*Dusp1*	Dual specificity phosphatase 1	1.45	−1.27
*Egr1*	Early growth response 1	1.57	−1.31
*Egr2*	Early growth response 2	2.79	−1.37
*Egr4*	Early growth response 4	1.53	−1.41
*Hspa1a*	Heat shock 70 kD protein 1A	2.12	−1.26
*Hspa1b*	Heat shock 70 kD protein 1B	1.84	−1.46
*Ier5*	Immediate early response 5	1.29	−1.17
*Junb*	Jun-B oncogene	1.50	−1.36
*Klf10*	Krueppel-like factor 10	1.36	−1.29
*Nr4a1*	Nuclear receptor subfamily 4, group A, member 1	1.60	−1.23
*Nr4a3*	Nuclear receptor subfamily 4, group A, member 3	1.39	−1.20
*Ptgs2*	Prostaglandin-endoperoxide synthase 2	1.36	−1.12
*Sik1*	SNF1-like kinase	1.76	−1.12

**Figure 8:**
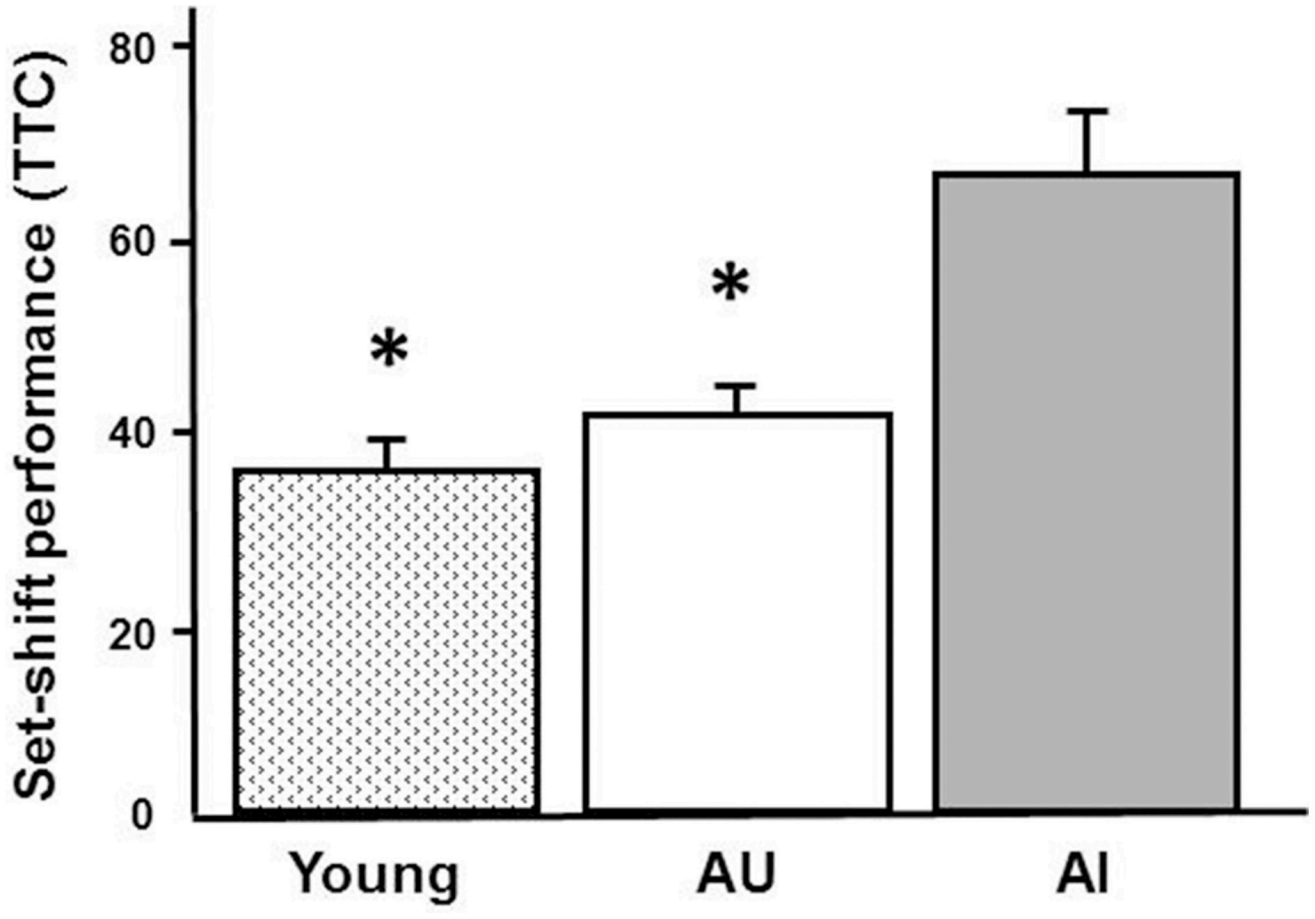
**Set shifting performance for animals used in RT-qPCR validations**. The animals were selected based on set shifting performance to insure group differences in behavior. The bars illustrate this difference as the mean + SEM TTC for young (*n* = 9) and aged animals classified as unimpaired (AU, *n* = 6) and impaired (AI, *n* = 6) on the set shift task. Asterisks indicate a significant (*p* < 0.05) difference relative to AI animals.

**Figure 9:**
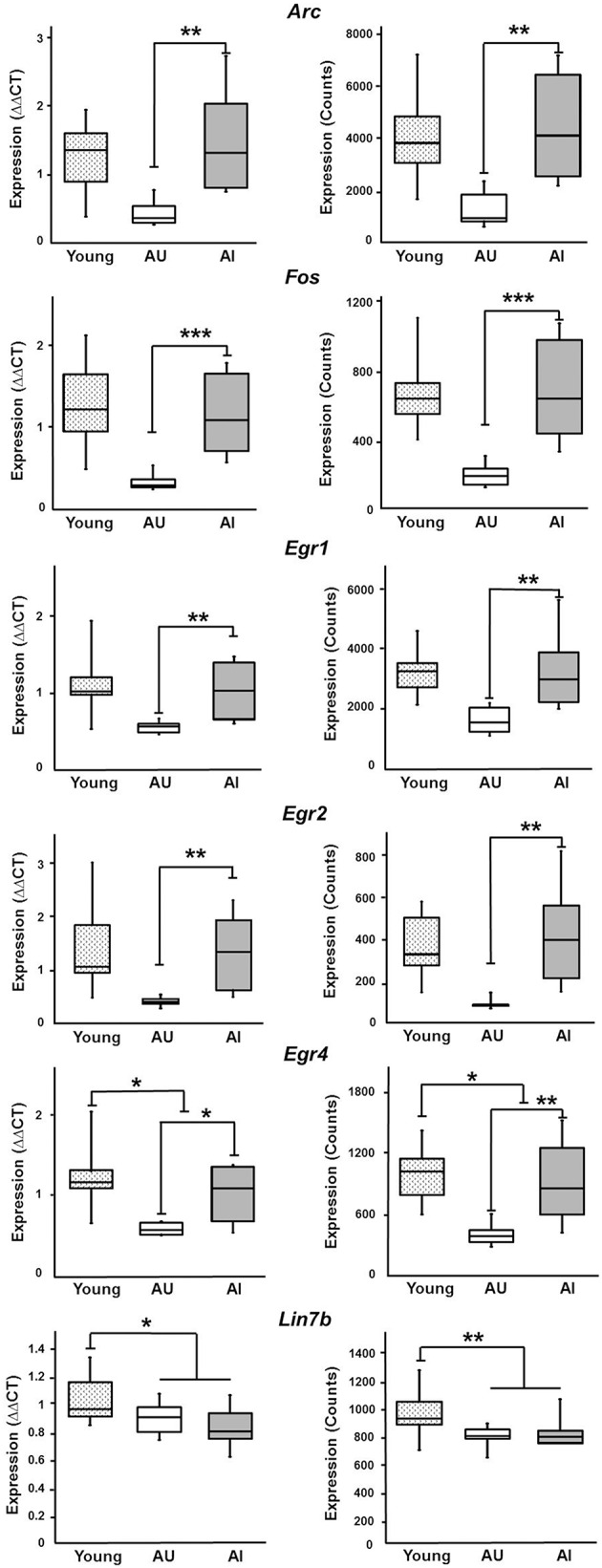
**Comparison between RT-qPCR and RNA-seq**. Six genes were selected for validation experiments using a subset of animals. Each panel provides the mPFC expression determined by RT-qPCR (left, ΔΔCT values) and RNA-seq (right, counts). Two-tailed *t*-tests confirmed increased expression of *Arc, Fos, Egr1, Egr2, and Egr4* in AI, relative to AU rats. Gene expression for young animals is provided for comparison to aged animals. For two genes, *Lin7b* and *Egr4*, age differences were confirmed (^***^p < 0.005, ^**^p < 0.025, ^*^p < 0.05).

In order to provide some validation of the findings, RT-qPCR was performed on 5 IEGs from a subset of young (*n* = 9), AI (*n* = 6), and AU (*n* = 6) animals. The animals were selected based on set shifting performance to insure group differences in behavior (Figure 8) and an ANOVA confirmed a group difference [*F*_(2, 18)_ = 17.15, *p* < 0.0001]. *Post-hoc* tests confirmed that AI animals exhibited an increase in the TTC relative to young and AU animals. Figure 9 shows a comparison of RT-qPCR results relative to the gene counts for the same genes and animals. The genes selected were IEGs that were increased in impaired animals (*Arc, Egr1, Egr2, Egr4, Fos*) and the comparisons (i.e., *t*-tests) were limited to confirmation of results observed for the whole data set. For both the RNA-seq and RT-qPCR measures of expression, *t*-tests indicated a difference in expression (*p* < 0.05) between the subset of AI and AU animals (Figure 9). In addition, *Lin7b* and *Egr4* were expected to decrease with age, which was confirmed for the RNA-seq and RT-qPCR using *t*-tests to compare the subset of young and aged animals (Figure 9).

## Discussion

Neuroimaging indicates that the pattern of cognitive decline is related to the structure or activity of specific brain regions (Grady et al., 2005; Persson et al., 2006; Dennis et al., 2008; Park and Reuter-Lorenz, 2009; Migo et al., 2016). Furthermore, altered white matter integrity could influence connectivity between brain regions (O'Sullivan et al., 2001; Pfefferbaum et al., 2005; Salat et al., 2005; Andrews-Hanna et al., 2007; Bennett et al., 2011; Lu et al., 2011; Borghesani et al., 2013). While similar biological processes were altered across regions with age, very few genes were similarly affected across regions. Dissimilarities may relate to regional differences in vulnerability. Furthermore, vulnerability to aging is influenced by environment and lifestyle such that food restriction to promote operant behavior as well as the training procedure, may have differentially modified aging processes across regions (Lee et al., 2000; Zeier et al., 2011). Nevertheless, RNA-seq profiles confirmed that brain aging is associated with biological processes that involve increased expression of immune/defense response genes and decreased mitochondria and neuronal/synaptic genes (Prolla, 2002; Blalock et al., 2003; Lu et al., 2004; Verbitsky et al., 2004; Bordner et al., 2011; VanGuilder et al., 2011; Zeier et al., 2011; Cribbs et al., 2012; Berchtold et al., 2013; Primiani et al., 2014).

Age-related differences in cortical transcription are observed across species possibly due to evolutionary constraints on aging, examination of disparate brain regions, or differences in the age range examined (Erraji-Benchekroun et al., 2005; Fraser et al., 2005; Loerch et al., 2008; Cribbs et al., 2012; Berchtold et al., 2013). The mPFC of rats is thought to be functionally related to the dorsolateral PFC in humans and monkeys (Dias et al., 1997; Birrell and Brown, 2000; Kesner and Churchwell, 2011). Similar to the PFC of humans, we found that genes linked to excitatory and inhibitory transmitter systems decline with age in the mPFC. Significantly, age-related changes in gene expression did not predict cognition.

An important contribution of the current research was the specificity of transcriptional changes that correlate with a cognitive function that depends on the mPFC. The mPFC contributes to cognitive flexibility and spatial learning (Churchwell et al., 2010); however, no correlation was observed between set shifting and acquisition of a spatial search strategy (Barense et al., 2002; Beas et al., 2013). Transcription in the mPFC reflected this distinction in that genes correlated with the discrimination index generally decreased expression (62%) in impaired animals, while a large proportion (73%) of genes associated with impaired set shifting exhibited increasing expression. Specificity of mPFC transcription was also reflected in the 3-fold increase in the number of genes that correlated with set shift performance relative to visual discrimination learning. Finally, many of the activity-related IEGs that increased in impaired animals, exhibit down regulation in the mPFC during aging; and down regulation of IEGs in other brain regions is associated with cognitive impairment and decreased responsiveness (Benloucif et al., 1997; Blalock et al., 2003; Rowe et al., 2007). The results indicate specificity of mPFC transcription with an age-related impairment of mPFC-dependent behavior.

Altered basal expression of PFC IEGs is observed across rat species that exhibit differences in executive function. Upregulation of neural activity and synaptic plasticity genes is observed for SHR rats, which exhibit impaired set shift behavior compared to Wistar-Kyoto rats (Qiu et al., 2010). Similarly, compared to successful aging of LOU/C/Jall rats, aging in Wistar rats is associated with working memory deficits and an increase in PFC expression of IEGs (*Arc, Egr2, Fos, Junb*, and *Nr4a1*; Paban et al., 2013). Differences in transcription across rodent strains may result from genetic polymorphisms. Thus, an important finding from the current study is that individual variability in cognition, within the same species, is associated with increased basal expression of mPFC IEGs indicating that increased expression is indicative of impaired cognitive flexibility during aging.

What mechanism could increase IEG expression in animals that delay set shift behavior? Differences in IEG expression could result from differences in epigenetic regulation of transcription during aging (Peleg et al., 2010; Hernandez et al., 2011). Indeed, set shifting behavior was associated with increased expression of genes involved in regulating histone deacetylase (*Bcor, Ccdc101, Dnajb5, Kdm6b*) and histone acetyltransferase (*Ing3*) activity. Alternatively, IEG expression is upregulated by increased neuronal activity (Ghosh et al., 1994; Guan et al., 2009; Rudenko et al., 2013). Impaired set shift behavior was correlated with increased expression of transcription factors of inhibitory neurons (*Dlx1, Npas1*) and genes linked to the strength of excitatory and inhibitory inputs (*Arc, Npas4*) suggesting altered synaptic plasticity and increased neural activity. Interestingly, an increase in frontal cortex neural activity is observed in older humans and may relate to performance of cognitive tasks (Rosano et al., 2005; Turner and Spreng, 2012; Maillet and Rajah, 2014).

One possible mechanism for impaired set shift performance and increased neuronal activity involves a decline in N-methyl-D-aspartate (NMDA) receptor function. The degree of mPFC NMDA receptor hypofunction is correlated with impaired attention starting in middle-age (Guidi et al., 2015). Furthermore, blockade of NMDA receptors in this region disrupts set shift behavior (Stefani and Moghaddam, 2005; Dalton et al., 2011). The mechanism appears to involve a shift in the balance of excitatory/inhibitory synaptic input since inhibition of NMDA receptors in the PFC decreases the activity of inhibitory interneurons and increases the discharge activity of pyramidal cells (Homayoun and Moghaddam, 2007). Thus, an age-related decline in NMDA receptor function may reduce inhibitory drive and increase expression of activity-related genes in pyramidal cells.

In summary, the results support the idea that aging is associated with an increase in expression of immune and defense response genes and a decline in synaptic and neural activity genes. Importantly, mPFC expression of IEGs related to neural activity and synaptic plasticity decline with age; however, expression is up regulated in aged animals that exhibit delayed set shift behavior. The mPFC transcriptional profile of impaired animals is in contrast to decreased IEG expression reported for the hippocampus and other brain regions during aging. The specificity of impairment on a mPFC-dependent task, associated with a particular mPFC transcriptional profile indicates that impaired executive function involves altered transcriptional regulation and neural activity/plasticity processes that are distinct from that described for impaired hippocampal function.

## Author contributions

TF, AK, LI designed experiment, AR, LI, AK, and BB performed research, TF and LI analyzed data, constructed figures, and wrote paper

### Conflict of interest statement

The authors declare that the research was conducted in the absence of any commercial or financial relationships that could be construed as a potential conflict of interest.
